# Complete Genome Sequence of *Arthrobacter* Phage ScienceWizSam

**DOI:** 10.1128/mra.00927-22

**Published:** 2022-11-07

**Authors:** Samantha Coplen, Charmaine Lopez-Davis, Hari Kotturi

**Affiliations:** a Department of Biology, University of Central Oklahoma, Edmond, Oklahoma, USA; Loyola University Chicago

## Abstract

Phage ScienceWizSam was isolated from soil using *Arthrobacter* sp. strain ATCC 21022. The phage genome is 58,217 bp with 96 open reading frames (ORFs). All of the ORFs are transcribed rightwards. Based on gene content similarity, ScienceWizSam is assigned to phage subcluster AU1.

## ANNOUNCEMENT

The genus *Arthrobacter* is a morphologically, phylogenetically, and physiologically diverse group of Gram-positive bacteria present in terrestrial and aquatic ecosystems. Multiple *Arthrobacter* strains are of human interest due to their potential applications in medicine, agriculture, and biotechnology (1). Many phages that infect *Arthrobacter* hosts have been isolated and characterized, providing insights into their genetic diversity (2). Here, we report on phage ScienceWizSam, which was isolated from a soil sample collected from a private backyard in Oklahoma City, OK (35.3828111N, 97.5514035W), using *Arthrobacter* sp. strain ATCC 21022 and standard procedures (3). Briefly, ScienceWizSam was isolated by washing the soil sample with peptone-yeast extract-calcium (PYCa) liquid medium followed by filtration with 0.22-μm-pore filter. The filtrate was inoculated with *Arthrobacter* sp. and incubated with shaking for 2 days at 28°C. The filtered culture was then plated in top agar with *Arthrobacter* sp., yielding phage ScienceWizSam, which forms clear plaques measuring 3 mm in diameter after 2 days at 28°C. ScienceWizSam was subsequently purified through three rounds of plating.

The Promega Wizard DNA cleanup kit was used to extract phage genomic DNA. Genomic libraries were generated at the University of Pittsburgh using the Ultra II FS kit (New England Biolabs). An Illumina MiSeq system (v.3 reagents) was used for sequencing the prepared libraries and yielded 1.3 million single-end 150-bp reads with an approximate shotgun coverage of 3,178. Newbler v.2.9 (4) and Consed v.29 (5) were used with default parameters for assembling the genome and assessing the quality of the assembly (6). The phage genome is 58,217 bp long with 50% GC content and genome termini composed of 3′ single-stranded overhangs (5′-CGCCGGCCT-3′). ScienceWizSam is assigned to phage cluster AU, subcluster AU1, based on gene content similarity of at least 35% to phages in the Actinobacteriophage database (7, 8).

The genome was annotated using DNAMaster v.5.23.6 (http://cobamide2.bio.pitt.edu/computer.htm), GeneMark v3.25 (9), Glimmer v3.02b (10), NCBI BLAST v.2.9.0 (11), Starterator v.1.2 (http://phages.wustl.edu/starterator), HHpred v.3.2.0 (12), ARAGORN v.1.2.41 (13), Phamerator (14), TMHMM v.2.0 (15), and SOSUI (16).

Genome annotation revealed that ScienceWizSam has 96 total open reading frames (ORFs), all of which are transcribed on the same strand and 39 of which could be assigned functions. Predicted structural and assembly genes occur on the left half of the genome, encoding terminase (gp8), a notable major capsid and protease fusion protein (gp14), a tape measure protein (gp28), major tail proteins (gp17 and gp19), and minor tail proteins (gp20, gp21, gp23, gp29, and gp30). Also encoded on the left half of the genome are HNH endonuclease (gp3) and endolysin (gp4). The right half of the genome encodes DNA metabolism functions, including DNA polymerase (gp57) and helicase (gp73) among others. No immunity repressor or integrase could be identified, suggesting ScienceWizSam is a lytic phage consistent with other subcluster AU1 phages. ScienceWizSam also lacks tRNA genes.

### Data availability.

The complete genome sequence of phage ScienceWizSam is available in GenBank under accession no. ON645338 with NCBI SRA accession no. SRX14485117.
